# Mechanical circulatory support as a cornerstone in advanced heart failure and transplantation

**DOI:** 10.3389/fcvm.2025.1569188

**Published:** 2025-10-24

**Authors:** Marta Braga, Ana Isabel Pinho, Ulrich Jorde, José González-Costello

**Affiliations:** ^1^ Department of Cardiology, Unidade de Saúde Local São João, Porto, Portugal; ^2^ Faculty of Medicine, University of Porto, Porto, Portugal; ^3^ Division of Cardiology, Montefiore Medical Center and Albert Einstein College of Medicine, Bronx, NY, United States; ^4^ Cardiology Department, Bio-Heart Cardiovascular Diseases Research Group, Bellvitge Biomedical Research Institute (IDIBELL), Bellvitge University Hospital, L'Hospitalet de Llobregat, Barcelona, Spain; ^5^ Department of Clinical Sciences, School of Medicine, University of Barcelona, Barcelona, Spain; ^6^ Centro de Investigación Biomédica en Red de Enfermedades Cardiovasculares (CIBERCV), Madrid, Spain

**Keywords:** temporary mechanical circulatory support, durable mechanical circulatory support, advanced heart failure, transplantation, bridge to transplant

## Abstract

Orthotopic heart transplantation remains the gold standard for managing selected patients with end-stage heart failure (HF) who are unresponsive to conventional therapies. Mechanical circulatory support (MCS), encompassing durable (dMCS) and temporary (tMCS) devices, has become a cornerstone in bridging patients to transplant (BTT) and also addressing the increasing burden of advanced HF with dMCS destination therapy. Each type of MCS offers distinct advantages tailored to specific patient needs and clinical scenarios. This review summarizes the features of MCS devices, their implications in clinical practice, and their impact on patient outcomes. Evidence demonstrates that dMCS, including the widely used durable left ventricular assist device HeartMate 3, significantly improves the prognosis of waitlisted patients and is associated with better post-transplant outcomes compared to tMCS when used as a BTT strategy. However, recent trends in allocation systems favor prioritizing tMCS-supported patients to improve outcomes for sicker individuals, underscoring the complexity of resource allocation. In this context, recent tMCs devices such as the Impella 5.5 have demonstrated promising early results as BTT, and ongoing larger studies with long-term follow-up will be crucial to better define their optimal indications and patient selection. Additional research is required to ascertain whether urgency-based models provide the most equitable distribution of resources while optimizing both pre- and post-transplant outcomes. Continued innovation in MCS technology, alongside the development of personalized treatment strategies, is vital to address the evolving needs of the growing advanced HF population. Future advancements should prioritize creating devices that are easier to implant, feature wireless power sources, and provide more physiological support, ultimately enhancing the care and outcomes of patients with advanced HF.

## 1 Introduction

Orthotopic heart transplantation (HT) is the standard of care for selected patients with end-stage heart failure (HF) refractory to medical management. HT enhances survival rates, quality of life, and the likelihood of returning to work, as long as patients are selected appropriately (1). The availability of donor hearts is the main challenge in HT, varying significantly across the world and influencing local transplant allocation criteria. Advances in selecting recipients and donors, as well as post-transplant management have led to a survival improvement of transplant recipients over time. Data from the International Society for Heart and Lung Transplantation (ISHLT) Registry indicate that the median survival after adult HT is 12.5 years, increasing to 14.8 years among patients who survive the first post-transplant year (2). Survival outcomes are influenced by primary diagnosis, recipient age, and donor characteristics. Patients transplanted for non-ischemic cardiomyopathy exhibit the highest 1-year survival rates. Individuals with congenital heart disease demonstrate superior long-term survival, conditional on surviving the early post-transplant period. Conversely, recipients with ischemic cardiomyopathy and those undergoing retransplantation tend to have the poorest long-term survival (2). The clinical stability of a patient before a HT is also a strong predictor of early post-transplant success (1). Critically ill patients often require mechanical circulatory support (MCS) to stabilize their condition, reassess their eligibility for transplantation, and await a suitable donor heart. In recent years, there has been an increase in the use of MCS devices in these patients, both short-term and long-term, acting as a direct bridge to transplant (BTT), as a bridge to candidacy (BTC) or even as bridge to bridge (BTB). In this article, we review the role of MCS in transplant candidates, highlighting their clinical indications, decision making process, and the impact on post-transplant outcomes and survival rates. Additionally, we explore the implications of MCS on HT allocation systems worldwide and investigate existing gaps and future directions in this evolving field.

## 2 Methods

### 2.1 Search strategy

The literature search was conducted in three electronic databases: MEDLINE (through PubMed), ClinicalTrials.gov and Cochrane Library. Published research was collected using combinations of terms, including “bridge to heart transplant”, “advanced heart failure”, “heart failure”, “cardiogenic shock”, “heart transplantation”, “mechanical circulatory support”, “temporary mechanical circulatory support”, “durable mechanical circulatory support”, “intra-aortic balloon pump”, “extracorporeal membrane oxygenation”, “percutaneous ventricular assist devices”, “surgical ventricular assist devices”, “total artificial heart” and related terms, as well as synonyms and variant spellings to broaden the search scope. MeSH terms and keywords were combined accordingly on the respective databases previously mentioned. Titles and abstracts of articles published from 1999 to May 2025 available in English were evaluated. Following this initial search, further articles were identified by manually examining the references of the retrieved studies.

### 2.2 Eligibility

Studies were included when the following general criteria were met: (1) observational studies, controlled trials, editorials, international reports, reviews, meta-analysis and systematic review articles describing outcomes of left, right and biventricular assist devices and/or HT, (2) reported data in adult patients, (3) studies published in English. Duplicate publications were identified and excluded. All non-human studies, abstracts, conference communications and individual case reports were excluded.

### 2.3 Study selection and data collection

Abstracts were screened for study eligibility and manuscripts were reviewed for data extraction by two reviewers. After the primary screening and data extraction, both authors performed quality control, which included verification of reasons for study inclusion and exclusion and verification of all extracted data. Discordant decisions were managed by discussion and consensus among additional authors as necessary. A comprehensive literature review was conducted on MCS strategies in cardiogenic shock (CS) and chronic advanced HF, including ventricular assist device implantation for specific etiologies, with a focus on bridging to transplantation.

## 3 Overview of MCS devices

**Figure 1:**
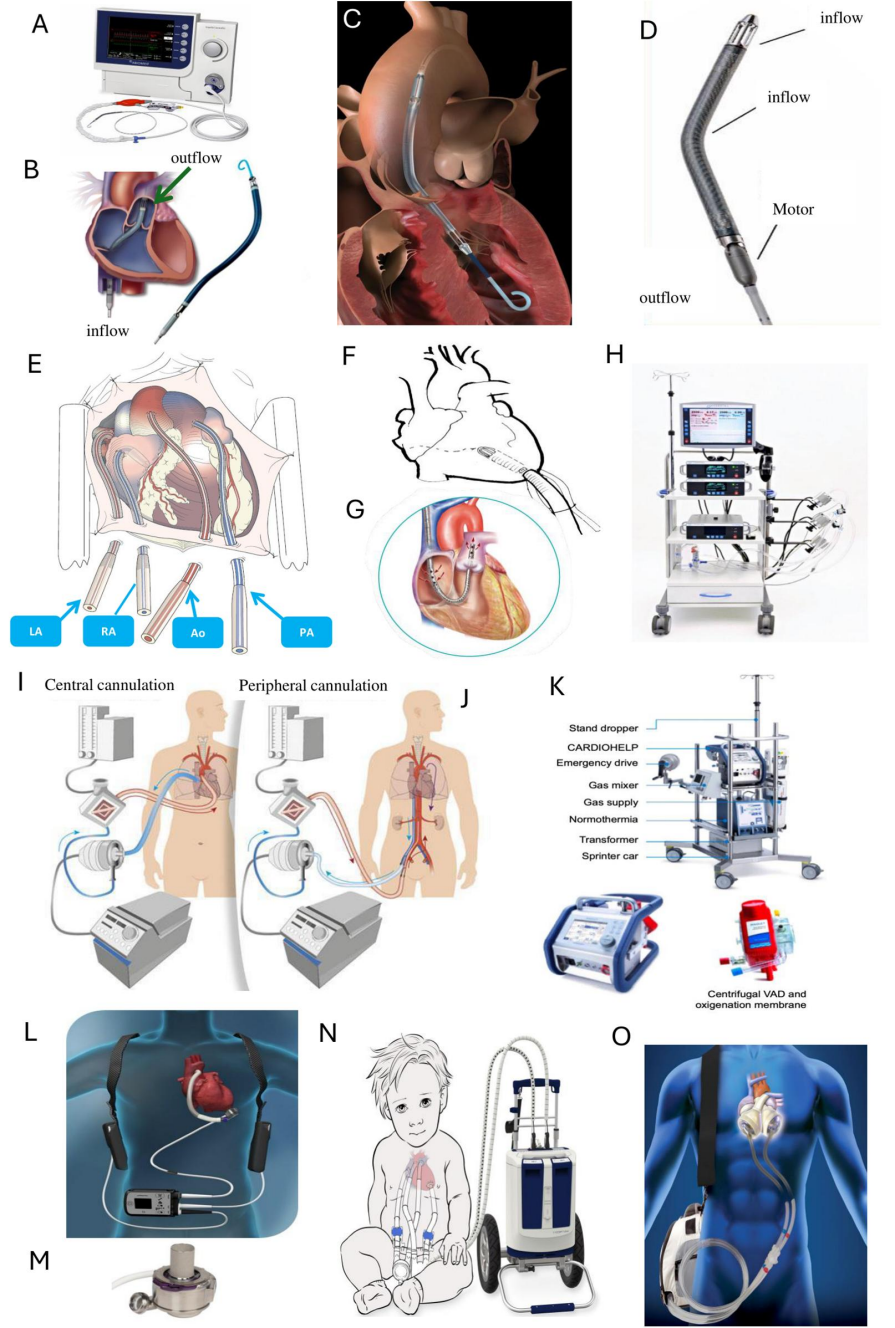
Examples of mechanical circulatory support (MCS) devices and configurations. Upper panel: Impella devices (Abiomed, Johnson & Johnson®, Massachusetts, USA) **(A)** Impella console, **(B)** Impella RP, **(C)** Impella CP, and **(D)** Impella 5.5. Middle upper panel: **(E)** Cannulation of the great vessels with Centrimag (Abbott®, Illinois, USA), **(F)** Cannulation of the left ventricle with Centrimag, **(G)** ProtekDuo (LivaNova®, London, UK), and **(H)** Centrimag console. Middle lower panel: **(I)** VA-ECMO with central cannulation, **(J)** VA-ECMO with peripheral cannulation, and **(K)** ECMO console and oxygenator. Lower panel: Durable mechanical circulatory support devices **(L)** Overview of the left ventricular assist device (LVAD) system, HeartMate 3 (Abbott®, Illinois, USA), **(M)** HeartMate 3 LVAD cannula, **(N)** Berlin Heart EXCOR® (Berlin Heart GmbH, Berlin Germany), and **(O)** Total Artificial Heart (SynCardia®, Tucson, USA). Ao, aorta; LA, left atrium; PA, pulmonary artery; RA, right atrium. Images provided by: **A–D**: Johnson and Johnson; **E**, **F**, **H**, **L**, **M**: Abbott; **G**: Palex; **I**, **J**: Hospital Universitari de Bellvitge; **K**: Gettinge; **N**: BerlinHeart; **O**: Mercé.

MCS devices are advanced technologies designed to assist or replace heart function in patients with severe cardiac conditions. These devices vary in design, functionality, and duration use. Several types of MCS devices are currently used in clinical practice (Figure 1), each tailored to specific patient needs and clinical scenarios.

Temporary MCS (tMCS) provides short-term hemodynamic support, lasting from hours to weeks, and can act as a bridge to recovery (BTR), decision-making, or transition to long-term options such as durable MCS (dMCS) or HT. tMCS offers multiple configurations for right, left, or biventricular support (Table 1). These options include fully percutaneous systems that access peripheral vessels via catheters or cannulas (intra-aortic balloon pump—IABP; Impella CP/RP, Abiomed, Johnson & Johnson®, Massachusetts, United States of America [USA]; TandemHeart, LivaNova®, London, United Kingdom [UK]; Venous-arterial extracorporeal membrane oxygenation—VA-ECMO), surgically implanted systems with centrally placed cannulas or grafts connected to external tMCS devices (Impella 5.5, Abiomed, Johnson & Johnson®, Massachusetts, USA; Centrimag, Abbott®, Illinois, USA) and hybrid models combining elements from both approaches. tMCS systems deliver both partial and full circulatory support, influencing factors such as myocardial oxygen demand, left ventricular unloading, and coronary artery perfusion. One of the distinguishing features of the latest Impella device, the Impella 5.5, is its ability to provide full left-sided cardiac support via axillar artery or ascending aorta access, enabling patient ambulation and supporting early rehabilitation, with implications on frailty reversal (3–5). tMCS devices can be combined to adapt the support to the specific requirements of the patient. For instance, ECPELLA pairs VA-ECMO with Impella to achieve left ventricular unloading (6). Similarly, VA-ECMO can be combined with an IABP. Other setups include BiPella for biventricular support (7), or the ProtekDuo (LivaNova®, London, UK) cannula combined with a centrifugal pump for right-sided support (8) along with Impella for left-sided support.

**Table 1:** Types and main characteristics of temporary mechanical circulatory support (3, 4, 8, 38, 54, 131, 150, 151).

Device characteristics	Temporary mechanical circulatory support						
	IABP	Impella (Abiomed, Johnson & Johnson®, Massachusetts, USA)			TandemHeart (LivaNova®, London, UK)	Centrimag (Abbott®, Illinois, USA)	VA-ECMO
		CP	5.5	RP			
Percutaneous	Yes	Yes	No	Yes	Yes	No	Yes^a^
Ventricular support	LV	LV	LV	RV	LV (but possible RV^b^ and BiV)	LV, RV or BiV	BiV
Placement	Descending Ao via FA^c^	LV via FA or AA	LV via AA or directly into ascending Ao	RA to PA via FV	Inflow: LA via FV. Outflow: Abdominal Ao via FA	Inflow: LV or RA Outflow: Ascending Ao or pulmonary artery	Inflow: RA via IJV or FV Outflow: Abdominal Ao via FA^a^
Hemodynamic support (L/min)	0.5–1.0	4.0	Up to 6.2	4.0	4.0	Up to 10	Up to 10
Mechanism of action	Counterpulsation balloon pump	Axial flow pump			Centrifugal extracorporeal pump	Centrifugal extracorporeal pump	Centrifugal extracorporeal pump + external membrane oxygenation
LV unloading	Yes	Yes		N/A	Yes	Yes	LV overloading
Myocardial oxygen demand	↓	↓			↔↓	↓	↔ or ↑
Systemic anticoagulation	Recommended	Yes			Yes	Yes	Yes
Durability of support	Days	Days	Weeks to a month^d^	Days to weeks	Days to weeks	Weeks	Days to weeks
Major contraindications	Severe aortic insufficiency, aortic dissection, peripheral vascular disease	Severe aortic stenosis, prosthetic aortic valve, LV thrombus, peripheral vascular disease, aortic dissection		Severe right valvular disease or prosthetic valves, PA disorders	Atrial thrombus, severe aortic insufficiency, aortic dissection, peripheral vascular disease	Unable to tolerate anticoagulation	Peripheral vascular disease, severe aortic insufficiency, aortic dissection
Major complications	Vascular injury, bleeding, hemolysis, thrombocytopenia, aortic dissection	Hemolysis, access complications (vascular and nerve injury), bleeding, valve injury, ventricular arrhythmia, device dislodgement, thrombosis			Vascular injury, thromboembolism, cardiac perforation, hemolysis	Thromboembolism or air embolism, bleeding, hemolysis, arrhythmias	Vascular injury, hemolysis, thromboembolism, Harlequin syndrome, increase in LV pressure.
Additional considerations	ECG/pulse-dependent, easy to insert and adjust, cath lab not mandatory, increases coronary flow	Impella CP proved to decrease all-cause mortality in STEMI-related cardiogenic shock. Impella 5.5 features Smart Assist technology for remote monitoring and real-time hemodynamic parameter calculation. Its tip lacks a pigtail shape, reducing thrombus accumulation risk and enabling longer implant duration. Enables ambulation.			Transseptal puncture required	Allows for patient mobility, possible minimally invasive insertion technique	Bedside insertion, full circulatory support even in resuscitation situations, may require strategies to decompress the LV

^a^ It can also be implanted centrally, with surgical approach: right atrium and aorta.

^b^ RV support uses an extracorporeal centrifugal-flow pump to deliver blood from the RA (inflow) to the main PA (outflow) via the ProtekDuo (LivaNova®, London, UK) cannula or using 2 cannulas via the FV and IJV.

^c^ It can be placed into the axillary or subclavian artery.

^d^ The Impella 5.5 was approved for up to 30 days of support in Europe and up to 14 days of support in the United States of America. However, single centre reports have documented successful use for up to 70 days.

AA, axillary artery; Ao, Aorta; BiV, biventricular; FA, femoral artery; FV, femoral vein; IABP, intra-aortic balloon pump; IJV, internal jugular vein; LA, left atrium; LV, left ventricle; N/A, not applicable; PA, pulmonary artery; RA, right atrium; RV, right ventricle; STEMI, ST elevation myocardial infarction; VA-ECMO, venous-arterial extracorporeal membrane oxygenation.

Regarding dMCS (Table 2), which provides support lasting from months to years, the approval of continuous flow devices for use as a BTT led to the rapid replacement of pulsatile technology by continuous flow pumps. This transition resulted in a threefold increase in the number of implants recorded in Interagency Registry for Mechanically Assisted Circulatory Support (INTERMACS) (9). Since the positive results of MOMENTUM 3 trial (10, 11), HeartMate 3 (Abbott®, Illinois, USA), a fully magnetically centrifugally levitated pump, has replaced previous devices and is currently the only approved dMCS for adult patients in most countries. This pump was specifically developed to minimize mechanical wear, blood shear stress, and stasis. By intermittently modulating pump speed to mimic natural pulsatility, it significantly reduces hemocompatibility-related adverse events such as strokes, bleeding, and thrombosis, when compared to Heartmate II (Abbott®, Illinois, USA) (10) and HVAD (Medtronic®, Minneapolis, USA) (12). All these dMCS, as well as the Jarvik 2000 (Jarvik Heart® Inc., NY, USA) (13), were specifically designed to provide support to the left ventricle (left ventricular assistance device—LVAD). However, these systems have been also used clinically, off-label, in right ventricular (right ventricular assist device—RVAD) or biventricular failure (biventricular assistance device—BiVAD) setting (8). Modifications for placement in the right chambers require adjustments to the standard implantation techniques used for durable LVAD. The outflow grafts are attached to the main pulmonary artery, and to avoid excessive flow to the lungs, durable RVAD flows must be limited (8, 14).

**Table 2:** Types and main characteristics of durable mechanical circulatory support (8, 38, 54, 131, 150, 151).

Device characteristics	Durable Mechanical Circulatory Support					
	HeartMate 3 (Abbott®, Illinois, USA)	HeartWare HVAD (Medtronic®, Minneapolis, USA)	Jarvik 2000 (Jarvik Heart® Inc., NY, USA)	EXCOR® (Berlin Heart GmbH, Berlin, Germany)	TAH (SynCardia® Systems, LLC, Tucson, USA)	Aeson® (Carmat TAH, Vélizy-Villacoublay, France)
Ventricular support	LV^a^	LV^a^	LV	BiV, LV or RV^a^	BiV	BiV
Placement	Inflow: LV apex Outflow: ascending Ao	Inflow: LV apex; Outflow: ascending Ao	Inflow: LV apex; Outflow: descending Ao	Inflow: LV apex or RA Outflow: ascending Ao or pulmonary artery	Replaces both ventricles	Replaces both ventricles
Hemodynamic support (L/min)	Up to 10	Up to 10	Up to 7	Up to 6.5	Up to 9.5	Up to 9.5
Mechanism of action	Intracorporeal fully magnetically levitated continuous flow centrifugal pump	Intracorporeal magnetic and hydrodynamic levitated continuous flow centrifugal pump	Intracorporeal continuous flow axial pump	Paracorporeal, pneumatic pulsatile flow pump	Intracorporeal pneumatic pulsatile flow pump	Intracorporeal biocompatible, sensor-based autoregulated pulsatile flow pump
LV unloading	Yes	Yes	Yes	Yes	N/A	N/A
Systemic anticoagulation	Yes	Yes + Aspirin 100 mg OD	Yes + Aspirin 100 mg OD	Yes + Aspirin 100 mg OD	Yes	Low dose anticoagulation + Aspirin 100 mg OD
Durability of support	Years	Years	Years	Months	Months to years	Months to years
Major specific contraindications	Inability to tolerate anticoagulation, right heart failure			Inability to tolerate anticoagulation, non-correctable anatomical issues	Inability to tolerate anticoagulation, body size incompatibility	Body size incompatibility
Main complications	RV failure, device failure, stroke, driveline infection, gastrointestinal bleeding	Ischemic and hemorrhagic stroke, RV failure, device failure, pump thrombosis, driveline infection, gastrointestinal bleeding	Pump failure, thrombosis, bleeding, endocarditis, driveline infection, neurological events, gastrointestinal bleeding	Thrombosis, stroke, infection, bleeding, device malfunction, limited mobility	Neurologic events, thrombosis, bleeding, driveline infection, device malfunction, renal or liver failure	Driveline infection, atelectasis left lower lobe, device-related complications, renal/liver failure
Additional considerations	Current standard of care for advanced HF; reduced thrombotic and hemolytic complications compared to earlier devices	Recently discontinued but still in use in many patients	The power cable for the pump exits through the retroauricular region reducing the risk of driveline infection	Currently, the only VAD specifically designed and approved for the pediatric population in the USA, Europe, and Canada.	The only TAH to receive full FDA approval; 2 sizes according to body surface area (50cc and 70 cc).	Bioprosthetic materials. Commercially available in Europe only.

^a^ Currently, there are no continuous flow centrifugal pump ventricular assist devices designed for right-sided use. However, commercially available left ventricular assist devices are being used in the RV position to support isolated RV or biventricular failure, even though they are designed for the systemic circulation.

Ao, aorta; BiV, biventricular; FDA, food and drug administration; HF, heart failure; LV, left ventricle; N/A, Not Applicable; OD, once daily; RA, right atrium; RV, right ventricle; TAH, total artificial heart; USA, United States of America; VAD, ventricular assist device.

Implanting two durable LVADs is a complex procedure and adds significant cost. Another option for dMCS is the Berlin Heart EXCOR® system (Berlin Heart GmbH, Berlin Germany), which is a pneumatically driven paracorporeal system that can provide univentricular or biventricular support (15). The EXCOR® device is now mainly used in pediatric patients due to its suitability for implantation in infants and children with small body sizes and its availability in multiple pump sizes. In adults, its use is limited because of the high risk of thromboembolic complications, pump dysfunction, and infections, requiring high surveillance and intensive clinical monitoring (16). The SynCardia® (SynCardia Systems, LLC, Tucson, USA) total artificial heart (TAH), the first Food and Drug Administration approved TAH (17), is designed for both in-hospital and out-of-hospital use as a BTT. However, its global adoption remains limited due to complexity of implantation and management (18). The Aeson®, Carmat TAH (Vélizy-Villacoublay, France), available only in Europe, is an electro-hydraulically powered biventricular pump made of bioprosthetic materials, engineered for patients with end-stage biventricular HF as a BTT and is presently undergoing clinical trials (19).

## 4 Clinical indications for MCS

MCS is essential for improving end-organ perfusion and reducing congestion in patients with severe HF who do not respond to standard treatments. The primary indications for tMCS implantation are CS due to acute myocardial infarction (AMI-CS) or acutely decompensated chronic HF of ischemic or non-ischemic etiology (HF-CS). Additional acute scenarios for tMCS use include fulminant myocarditis, stress-induced cardiomyopathy, peripartum cardiomyopathy, refractory arrhythmias and post-cardiotomy complications. In recent years, VA-ECMO has been employed following cardiac arrest for resuscitation purposes (20).

For long-term support, the 2023 updated ISHLT guidelines (9) recommend considering dMCS for patients with advanced HF symptoms (New York Heart Association functional class IIIB-IV) refractory to maximal medical management, inotrope dependent or on temporary support. In such cases, dMCS may serve as a direct BTT. When immediate transplantation is not possible, dMCS can improve transplant eligibility (BTC) or be used as a permanent solution for patients who are ineligible for transplant (destination therapy—DT). Guidelines also highlight recent onset nonischemic dilated cardiomyopathy unresponsive to optimal medical therapy as an indication for dMCS as a BTR, focusing on neurohormonal modulation and monitoring recovery of left ventricular function to evaluate candidacy for dMCS explant or decommission before considering HT (21).

## 5 Management of CS and chronic advanced HF and MCS decision-making

In recent years, there have been significant advancements in the understanding and management of CS. However, in-hospital mortality remains at approximately 50% for CS patients despite improved pharmacological and device-based strategies, timely revascularization, and advances in intensive care (22–28). The decision to initiate MCS therapy often depends on multiple factors including the severity of symptoms, underlying comorbidities, potential for cardiac and end-organ function recovery, eligibility and availability of HT/dMCS, clinical profile (acute vs. decompensated chronic disease, univentricular vs. biventricular failure) and local resources. The process should be guided promptly by a multidisciplinary CS team, comprising an intensivist, interventional cardiologist, HF cardiologist, and cardiac surgeon, as this collaborative approach has been shown to significantly improve CS patients' outcomes (29, 30).

The updated Society for Cardiac Angiography and Intervention (SCAI) classification (Table 3) provides a framework for guiding clinical management of CS and determining the optimal timing for tMCS initiation based on shock severity (26, 31–33). Early intervention is recommended for patients in advanced CS (SCAI stage C or worse), emphasizing circulatory stabilization and consideration of available tMCS devices (22, 25). Retrospective data indicates that utilizing complete pulmonary artery catheter (PAC)-derived hemodynamic information before initiating MCS is associated with improved survival in CS patients (34). Randomized trials may provide contemporary data regarding the role of PACs in CS (ClinicalTrials.gov: NCT05485376).

**Table 3:** Representation of interagency registry for mechanically assisted circulatory support (INTERMACS) and society for cardiovascular angiography and interventions (SCAI) classifications.

INTERMACS Profile	Description	SCAI Shock Stage	Description	Comments
7	Advanced New York Heart Association class III	A	At risk for cardiogenic shock	SCAI A generally corresponds to less severe INTERMACS profiles (4–7), representing patients at risk of shock.
6	Exertion limited			
5	Exertion intolerant, housebound			
4	Resting symptoms on oral therapy			
3	Stable but inotrope dependent	B	Beginning shock	SCAI B could align with INTERMACS 3, where patients require inotropic support but are relatively stable.
2	Progressive decline on inotropes	C	Classic cardiogenic shock	SCAI C to E correlate with the most severe INTERMACS profiles (1–2), representing declining patients and those in extreme shock.
1	Critical cardiogenic shock	D	Deteriorating	
		E	Extremis	

Both classifications help stratify patients for risk assessment and guide treatment decisions in advanced heart failure and cardiogenic shock. SCAI Shock Classification: Stages A to E represent increasing severity of cardiogenic shock. Stage C and above indicate the presence of hypoperfusion. INTERMACS Classification: Profiles 7 to 1 represent decreasing stability in advanced heart failure. Lower profiles are associated with higher risk of adverse outcomes. It's important to note that these classifications were developed for different purposes and don't perfectly align, but there are some general correlations we attempted to draw, as shown in the table. These correlations are approximate, as individual patient presentations can vary and may not fit neatly into these categories.

CS results from various cardiovascular conditions, most commonly AMI-CS and HF-CS. Currently, HF-CS accounts for over 50% of all CS cases (22, 25, 27, 33, 35).

### 5.1 The role of MCS in AMI-CS

AMI-CS typically presents abruptly in patients without previous history of HF (36) and early revascularization is the most evidence-supported intervention (37). Routine tMCS use is not currently recommended unless shock severity warrants it. In cases where shock is present at the time of revascularization, tMCS devices may be deployed before percutaneous coronary intervention to stabilize the patient and enable coronary revascularization (38). Although widely used, IABP did not show clear clinical benefits in AMI-CS patients in randomized trials (39, 40). Similarly, routine use of VA-ECMO in these patients failed to demonstrate clinical benefit in the ECLS-SHOCK trial and led to higher rates of bleeding and vascular complications (41, 42). Despite these limitations, VA-ECMO is still used in many cases of SCAI stages D and E CS due to its ease of deployment and ability to provide full biventricular hemodynamic support (36). In contrast, recent findings from the DanGer Shock trial (43) showed that routine use of Impella CP microaxial flow pump, when combined with standard care, significantly reduced 180-day mortality in established ST elevation AMI-CS. Impella CP was placed before revascularization in about half of patients in the device arm. Its use was also associated with increased adverse events, such as severe bleeding, limb ischemia, renal replacement therapy and hemolysis. Impella 5.5 has been increasingly utilized as a salvage therapy for AMI-CS patients with refractory shock due to left ventricular failure, owing to the higher level of cardiac support it provides compared to its device predecessors (3). Given the need for surgical implantation, in the acute setting, Impella 5.5 can be implanted after initial clinical stabilization or reserved as an option to escalate left-sided support or even used in conjunction with VA-ECMO to unload the left ventricle (ECPELLA) and subsequently facilitate VA-ECMO weaning (44). The absence of controlled trials limits definitive conclusions about its role, and a properly designed prospective study is warranted to clarify its efficacy and optimal timing in this population.

Improved outcomes have been reported in cardiology-led coronary care units, likely reflecting higher rates of timely revascularization (45). However, in cases of large myocardial infarction, patients may not achieve sufficient early post-infarction remodeling and may deteriorate before later-phase recovery can occur. tMCS may facilitate myocardial recovery, particularly with devices capable of unloading the left ventricle while providing full left-sided support—such as the Impella 5.5. By supporting the heart through the early remodeling phase, these devices may help the native myocardium better tolerate increased wall stress, potentially enabling successful device weaning (5, 46). While additional studies have demonstrated left ventricular recovery with other tMCS devices, including VA-ECMO alone (47), left ventricular unloading appears to play a key role in promoting myocardial recovery (48).

Based on current evidence, Impella CP appears to be the most appropriate first-line option for patients with AMI-CS refractory to medical treatment (SCAI stage C or higher) and left ventricular dysfunction, provided there are no contraindications (43). For patients with advanced CS stage and critically low cardiac output, VA-ECMO should be considered. Impella 5.5 can be an option in patients who fail initial support with percutaneous devices—particularly when myocardial recovery is anticipated—or in those who are candidates to HT/dMCS, in whom maintaining ambulation is crucial.

In cases of isolated primary right ventricular failure associated with AMI-CS, percutaneous options such as the Impella RP or a centrifugal pump with the ProtekDuo cannula are potential first-line tMCS strategies (8, 38, 49–51). If these are unavailable, a surgically implanted RVAD Centrimag or percutaneous VA-ECMO in case of biventricular dysfunction may also be a viable alternative (8, 52, 53).

When myocardial recovery fails to occur, definitive therapies such as HT or dMCS, as a BTC or as DT, should be considered.

### 5.2 The role of MCS in HF-CS and chronic advanced HF

Regarding HF-CS, it is often considered part of a continuum of chronic advanced HF rather than a clearly distinct clinical entity. Reversible and transient factors of decompensation (e.g., arrhythmia) that may contribute to the development of shock should be rapidly assessed and corrected (22, 35, 54). The chronically dysfunctional, and often enlarged, left ventricle in advanced HF is under elevated filling pressures, which rise even further with the onset of shock. These patients require rapid volume assessment, with management aimed at reducing congestion through diuresis or ultrafiltration and improving cardiac output using inotropes. Neurohormonal antagonists and beta-blockers should be avoided, while short-acting intravenous vasodilators such as nitroprusside may be considered in patients without severe hypotension (22, 54). As patient´s clinical status deteriorates, intravenous vasopressors should be initiated. If pharmacological interventions fail, afterload reduction and ventricular decongestion can be achieved with tMCS (54), and early tMCS use should be considered either as bridge to decision, BTR, BTT or dMCS. Given the lack of robust comparative data, available institutional resources and operator expertise remain pivotal in selecting the appropriate tMCS device in HF-CS. Temporary percutaneous LVAD should be the preferred initial choice for left ventricular dysfunction in HF-CS, guided by the SCAI stage and the level of hemodynamic support provided by the device, especially as a BTT or bridge to dMCS. Similarly to its use in AMI-CS, VA-ECMO should be reserved for those patients with advanced CS and/or in case of biventricular dysfunction, particularly when combined with a temporary LVAD capable of unloading left ventricle (36). As previously mentioned, these devices have also been employed as a BTR, with encouraging outcomes in patients with HF-CS, as documented in other studies (46–48, 55).

Management of chronic advanced HF has improved in the last decades with new therapies and monitoring tools; however, HF with reduced ejection fraction remains a progressive condition, and patients who are unresponsive to optimal therapy face worsening symptoms, decreased quality of life, and higher mortality. Specialized advanced HF teams are essential in providing regular follow-up, conducting risk assessments, and initiating early treatment discussions. Their early involvement can prevent severe clinical deterioration and facilitate timely decision-making regarding MCS or urgent transplantation when patients' conditions worsen. Risk calculators (Seattle HF Model, HF Survival Score) and cardiopulmonary stress testing aid in identifying high-risk patients who should be referred for advanced HF therapies (9, 20).

The INTERMACS classification system stratifies patients with advanced HF based on clinical severity, guiding management (56) and predicting outcomes after MCS implantation (57, 58) (see Table 3). INTERMACS 1–2 patients, who present with CS (HF-CS) and severe symptoms, are often considered ideal candidates for tMCS as a first step (20, 54, 56, 59), as previously discussed. dMCS should be considered if the patient cannot be weaned from tMCS but still has the potential for meaningful recovery of end-organ function and quality of life, and there is no evidence of irreversible end-organ damage. dMCS should also be considered for stable but inotrope-dependent (INTERMACS 3), who face high mortality with continued medical management (9). Repetitive doses of levosimendan are commonly used in the ambulatory setting as BTT, since intravenous administration of intermittent doses of levosimendan in outpatients with advanced HF has been shown to be safe and effective in reducing HF-related hospitalizations (60–62). The optimal duration of this approach and the ideal timing for dMCS implantation while awaiting a HT remains uncertain, although dMCS is associated with better outcomes in patients requiring inotropic support for more than one year (63). An as-treated analysis of the ROADMAP study (64, 65) showed that patients in INTERMACS profile 4 benefited from dMCS therapy with improved survival, functional status, quality of life, and reduced depression compared to optimal medical therapy, despite higher rates of adverse events in the first year. Conversely, profiles 5–7 did not show similar benefits, and current evidence does not support routine dMCS use in these patients (9, 66). Nevertheless, it may be considered after individual assessment in high-risk patients, with recurrent hospitalizations, progressive end-organ failure, refractory congestion, inability to perform cardiopulmonary stress test or peak oxygen consumption <12 ml/min/kg (or <50% of expected value) as BTT or DT (54). Given the better outcomes provided by HeartMate 3, further randomized comparative trials are necessary to confirm the role of dMCS in patients in INTERMACS >4 and potentially redefine treatment recommendations. In parallel, emerging data highlight a distinct subgroup of patients in whom durable LVAD support may facilitate meaningful myocardial recovery, offering an alternative therapeutic pathway beyond traditional BTT or DT strategies. Predictive tools such as the INTERMACS Cardiac Recovery Score help identify candidates for successful explantation, emphasizing the need for standardized protocols and optimized medical therapy during support (67, 68).

For patients who experience clinical deterioration and are not eligible for advanced HF therapies, a multidisciplinary team should discuss end-of-life options, including comfort measures and palliative care, while providing support to both patients and caregivers.

### 5.3 Support strategies in HT candidates

In cases where recovery from CS or chronic advanced HF is not achieved, and conventional therapies fail to provide adequate support, MCS becomes a crucial bridge. As mentioned before, MCS can serve as a lifeline in several ways: directly bridging patients to HT, facilitating their progression to candidacy for transplant, or, in some cases, supporting them through multiple stages of intervention with further MCS devices. For certain patients initially bridged, dMCS may ultimately serve as a DT, providing long-term support in the absence of ongoing transplant eligibility.

#### 5.3.1 Direct BTT

While urgent HT listing is an option in many countries, their appropriateness is increasingly being questioned. Data from the Spanish National Heart Transplant Registry indicate that patients listed urgently, particularly those with severe CS or progressive clinical decline despite treatment, experience the highest risks of primary graft failure, need for dialysis, and in-hospital mortality following HT (69). For these critically ill patients, stabilization with tMCS as a BTT offers a safer alternative to immediate transplantation, provided that multi-organ dysfunction has been resolved, as indicated by markers such as normalized lactate levels. Studies have shown that this strategy improves post-transplant outcomes (70). In this context, some transplant organizations have developed specific criteria to assess the absence of multi-organ failure (71).

Durable devices can support patients for extended periods, which is critical given the growing mismatch between donor organ availability and transplant demand. By offering a stable and sustainable solution, dMCS bridges the gap for patients who might otherwise decompensate while on the waiting list (72).

In clinical practice, the choice between tMCS and dMCS for direct bridging is dictated by the severity of the patient's condition, anticipated donor availability, and individual risk profiles. Integration of advanced decision-making algorithms and multidisciplinary care teams further enhances the effectiveness of these interventions.

#### 5.3.2 BTC

In patients with CS and associated multi-organ dysfunction, tMCS can play a critical role in reversing acute end-organ dysfunction. When initiated early, tMCS contributes to pre-transplant optimization by promoting renal function improvement (73–76) and, in selected cases, improving pulmonary hemodynamics, both key determinants of HT candidacy. Left ventricular unloading devices, such as the Impella 5.5, have been shown to reduce pulmonary capillary wedge pressure, pulmonary vascular resistance, and estimated right ventricular afterload (73, 74, 77, 78). These hemodynamic improvements not only stabilize the patient clinically but also allow a more accurate and comprehensive evaluation of transplant eligibility. dMCS devices can prolong this support, enabling long-term hemodynamic stabilization and functional rehabilitation. While the majority of recipients receive these devices as a BTT, only about half are listed for transplantation at the time of implantation (9). For those not initially listed, transplantation remains the ultimate goal, though various factors often render them ineligible at the onset. With dMCS therapy, some patients may achieve resolution or improvement in conditions such as pulmonary hypertension, renal dysfunction, or obesity, thereby enhancing their eligibility for transplantation. Additionally, for individuals with active drug abuse, uncertain social status, including insufficient psychosocial support or unresolved compliance concerns, dMCS also provides a window to address these factors. Nevertheless, the emergence of new complications during support or persistence of unresolved conditions, potentially precludes eligibility for transplantation and may change the indication of dMCS to DT.

#### 5.5.3 BTB

Since tMCS devices are not typically intended for long-term use, many patients require transition to more durable forms of support after a period of time due to complications or failure to achieve recovery. Patients who are clinically stabilized on tMCS but cannot be weaned from support and are not currently candidates for HT or for whom a donor heart is unavailable, a transition to dMCS can be an option as a BTC or BTT. On the other hand, patients who are not candidates for HT can use tMCS as a bridge to dMCS for DT (9). The selection of the appropriate dMCS device—whether durable LVAD, RVAD, BiVAD, or TAH—depends on several clinical factors, including the underlying etiology of CS, the type of tMCS used, the patient's hemodynamic profile (left, right or biventricular failure), candidacy for HT and institutional preferences and availability.

**Figure 2:**
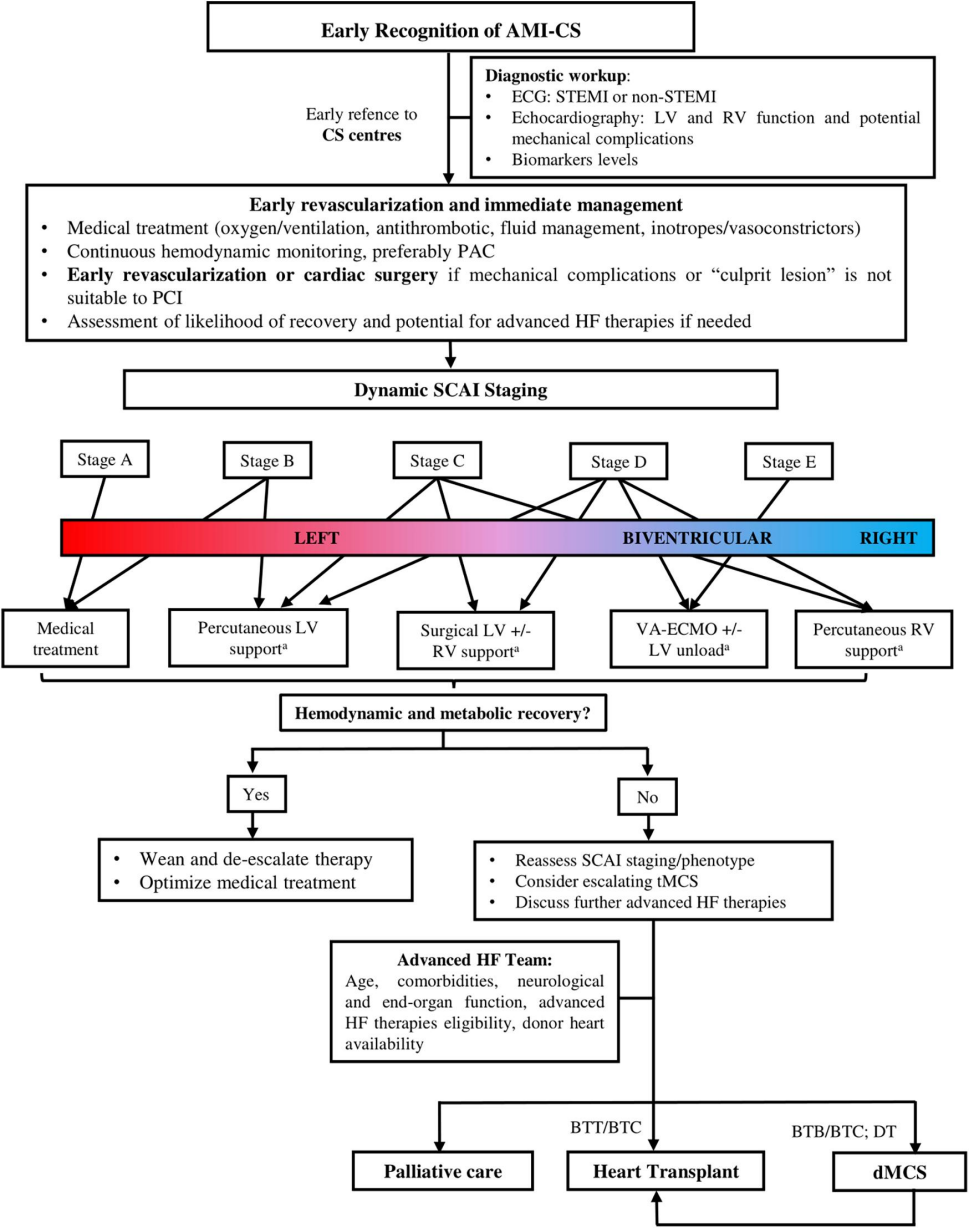
Algorithm for management of patients with cardiogenic shock related to acute myocardial infarction (AMI-CS). Legend: a. Device selection depends on availability and institutional expertise. AMI-CS, acute myocardial infarction related cardiogenic shock; BTB, bridge to bridge; BTC, bridge to candidacy; BTT, bridge to transplant; CS, cardiogenic shock; dMSC, durable mechanical circulatory support; DT, destiny therapy; HF, heart failure; IABP, intra-aortic balloon pump; LV, left ventricle; PAC, pulmonary artery catheter; RV, right ventricle; SCAI, Society for Cardiovascular Angiography and Interventions; STEMI, ST elevation myocardial infarction, tMSC, temporary mechanical circulatory support; VA-ECMO, arterial-venous extracorporeal membrane oxygenation.

**Figure 3:**
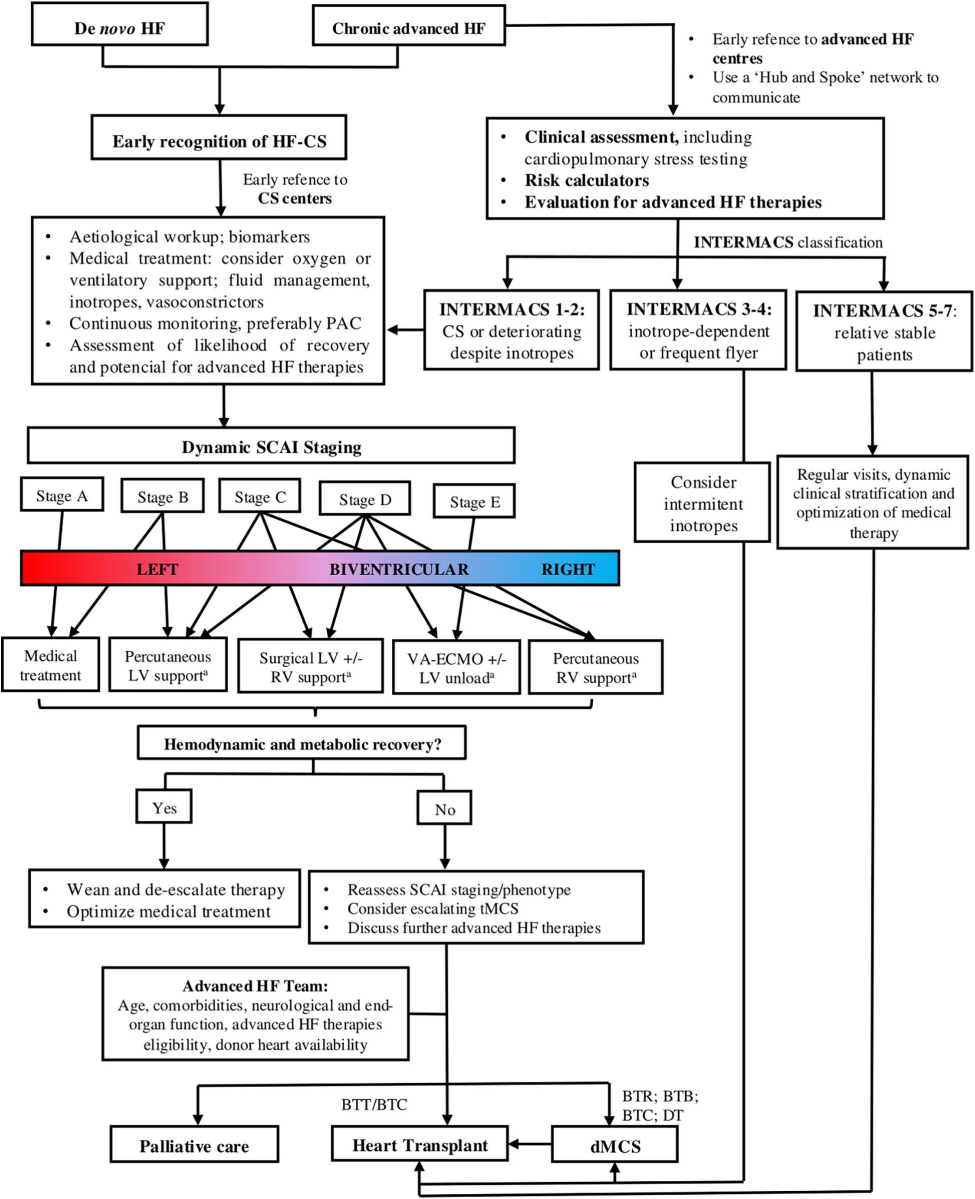
Algorithm for management of patients with advanced heart failure and cardiogenic shock related to HF (HF-CS). Legend: a. Device selection depends on availability and institutional expertise. BTB, bridge to bridge; BTC, bridge to candidacy; BTR, bridge to recovery; BTT, bridge to transplant; CS, cardiogenic shock; dMSC, durable mechanical circulatory support; DT, destiny therapy; HF, heart failure; HF-CS, heart failure related cardiogenic shock; INTERMACS, Interagency Registry for Mechanically Assisted Circulatory Support; LV, left ventricle; PAC, pulmonary artery catheter; RV, right ventricle; SCAI, Society for Cardiovascular Angiography and Interventions; tMCS, temporary mechanical circulatory support.

An algorithmic approach, as proposed by the authors in Figures 2, 3, can help guide clinical management of CS and chronic advanced HF, incorporating factors such as the patient's hemodynamic status, clinical trajectory and SCAI and/or INTERMACS classifications. This algorithm can be further adapted based on the country-specific resources, including HT waiting times, availability of donor organs, and experience with device implantation.

## 6 Post-transplant outcomes in patients with previous MCS

Survival outcomes for patients on MCS are multilayered, influenced not only by the type of support employed but also by the incidence of adverse clinical events while on support and patient-specific factors such as age, comorbidities, and overall functional status.

In patients supported with temporary devices, determining the optimal timing for HT constitutes a significant challenge. It requires balancing sufficient time to allow for recovery from end-organ dysfunction against avoiding prolonged waiting periods that increase the risk of adverse events related to prolonged use of tMCS (79). Although many complications of tMCS, such as infection, bleeding, thrombosis, and vascular injury, are treatable, they may imply a temporary contraindications to HT, increase mortality on the waiting list (80) and potentially compromise the success of the HT surgery (81, 82).

A 16-year analysis of the National Inpatient Sample of United States cohort of 6,892 patients who received an orthotopic heart transplant found improved outcomes over time of patients supported by tMCS before HT. Duration of tMCS support did not independently affect mortality. However, it was noted that the rate of post-transplant complications such as stroke and renal failure remained significantly higher in patients who received tMCS compared to patients without MCS (83).

Among tMCS bridging modalities, VA-ECMO has been linked to a higher incidence of adverse clinical events and increased early mortality post-transplant compared to other tMCS devices (81, 82, 84–87). In a cohort of 1,036 patients listed for emergency HT while on tMCS from 2010 to 2020, Barge-Caballero and colleagues found 1-year post-transplant survival of 67.8% in VA-ECMO group, lower than other tMCS, including IABP, Impella devices and LV/RV/BiV Centrimag support (79.4%, 84.9%, 74.4–79.9%, respectively, log rank *p* = 0.001) (82). After multivariate adjustment, preoperative bridging with VA-ECMO remained an independent predictor for post-transplant mortality (hazard ratio [HR] 1.71; 95% confidence interval [CI] 1.15–2.53, *p* = 0.008). In addition to these post-transplant concerns, VA-ECMO has also been associated to inferior waitlist outcomes. In a study by Moonsamy et al. (85), bridging with VA-ECMO was independently associated with a 2.4-fold increased hazard of death while awaiting transplantation compared to Centrimag support (HR: 2.40; 95% CI: 1.44–4.01; *p* = 0.001). However, in a subsequent sensitivity analysis evaluating 5-year survival conditional on surviving the first-year post-transplant, the authors found no significant differences between the various bridging strategies, likely due to the disproportionately higher mortality occurring within the first year in VA-ECMO group. These poorer outcomes observed in patients on VA-ECMO can be partly attributed to their typically lower INTERMACS profile score prior to tMCS implantation (88). Additionally, this form of support has been associated with several adverse physiological effects, including platelet dysfunction, an exaggerated systemic inflammatory response, increased left ventricular afterload, hydrostatic pulmonary edema, and direct pulmonary injury, which may complicate early postoperative extubation (89). A recent retrospective analysis of patients bridged to HT with VA-ECMO in 16 Spanish centers has shown that preoperative left ventricular unloading (using IABP in 84.2% of the cases) was independently associated with improved 1-year post-transplant survival (74.4% in the LV unloading group vs. 59.8% in the control group; adjusted 1-year mortality HR: 0.50; 95% CI: 0.32–0.78; *p* = 0.003) (90). Furthermore, patients on VA-ECMO often have limited time for end-organ recovery and are unable to undergo a comprehensive pre-transplant assessment, including psychiatric evaluation, informed consent, social assessment, medical compliance evaluation, and physical rehabilitation, which are more feasible with longer-term and/or ambulatory devices.

The Impella 5.5 provides full left ventricular support and achieves active ventricular unloading, reducing filling pressures and improving myocardial perfusion—physiological effects that may influence post-transplant outcomes (91). Since its approval, the Impella 5.5 has been increasingly used as a BTT. Using data from the United Network for Organ Sharing (UNOS) registry, Cevasco et al. reported a 1-year post-transplant survival of 89.5%, indicating favorable outcomes (92). In a separate analysis, Hill et al. found a comparable 1-year survival rate of 94.6% among patients supported with the Impella 5.5, reinforcing the consistency of these results (93). In a single-center retrospective study involving 43 patients (94), those undergoing HT who were bridged with the Impella 5.5 device required significantly less intraoperative transfusion of cryoprecipitate, autologous blood salvage, and platelets compared to patients bridged with a durable LVAD; although the study did not adjust for potential confounding variables such as baseline coagulation profiles and preoperative anticoagulation management, and lacked long-term outcome data, these findings suggest that the temporary and less invasive nature of the Impella 5.5 may reduce surgical complexity and bleeding risks during device explantation. As previously mentioned, the Impella 5.5 facilitates pretransplant rehabilitation while providing full hemodynamic support for several weeks—an approach that is critical for preserving muscle strength, preventing pressure injuries, and potentially reducing hospital length of stay, with positive implications for post-transplant results (5, 73, 95, 96). A retrospective cohort study (*n* = 65) showed that the use of Impella 5.5 enabled participation in pretransplant rehabilitation protocols, which were associated with improved post-transplant outcomes. Patients demonstrated higher standardized Activity Measure for Post-Acute Care Basic Mobility scores (adjusted coefficient 0.3, *p* = 0.04), greater improvements during rehabilitation (adjusted coefficient 0.35, *p* = 0.04), and more days alive outside the hospital within 30 days post-transplant (median 15 days). The ability to engage in extended rehabilitation while stabilized on Impella 5.5 highlights its role in optimizing functional recovery prior to transplantation (97). Of note, axillary artery access was associated with insertion site complications, such as access bleeding, arm ischemia and transient deficiency of the brachiocephalic plexus (see Table 1) (5).

As experience with the latest temporary devices grows, accumulating evidence supports that bridging with a durable LVAD is associated with improved post-transplant survival, especially when compared to VA-ECMO supported strategy. Data from ISHLT Registry (86) showed that bridging with tMCS, including VA-ECMO (HR: 3.79; 95% CI: 2.69–5.34; *p* < 0.001) and Impella/TandemHeart (HR: 1.83; 95% CI: 1.09–3.08; *p* = 0.02), was independently associated with a higher risk of 1-year post-transplant mortality compared to patients supported by durable LVADs. Similarly, in an analysis of the UNOS registry, Karamlou and colleagues demonstrated that patients supported by durable LVAD exhibit better post-transplant survival at 5-years compared to those supported by other forms of MCS, which included IABP and VA-ECMO (adjusted HR: 0.71; 95% CI: 0.59–0.84; *p* < 0.001) (98). In a separate analysis of the same registry including 26,918 recipients, another group found that over the first 16.7 years post-transplant, the estimated adjusted restricted mean survival time (defined as the maximum observed time from transplant to death) was 16.5 months (99% CI: 13.9–19.2), longer in patients bridged with durable LVADs compared to those bridged with VA-ECMO (87).

Although a survivor bias likely contributed (87), these findings highlight the advantages of dMCS support as a BTT, particularly in light of the significant improvements in post-transplant outcomes for durable LVAD patients over time (72). Earlier-generation devices, such as the HeartMate II and HVAD, were associated with higher 90-days post-transplant mortality but showed comparable long-term survival rates to *de novo* HT (82.6% vs. 83.4% at unadjusted 5-year survival; *p* = 0.15). Additionally, functional status, unadjusted rates of hospital readmission and graft rejection were similar at 1, 2 and 5 years (99). A similar finding was reported in another study (100), where 1-year post-transplant survival was 92.8% among medically bridged patients compared to 90.5% in patients supported with durable LVADs (log-rank *p* < 0.001). However, this difference was no longer evident at 5 years post-transplant, with survival rates of 78.9% in medically managed patients and 78.0% in those bridged to transplant with LVADs (*p* = 0.659), indicating no significant difference in long-term risk conditional on survival to 1 year. The median duration of LVAD support was 213 days (interquartile range, 121–377 days). Compared with patients who received <6 months of support, those with longer durations of support experienced higher mortality within the first year after HT (100). Early mortality observed in durable LVAD-supported patients has been associated to factors such as re-sternotomy, prolonged exposure to non-physiological flow, postoperative vasoplegia, different listing statuses, extended waiting times, and complications inherent to durable support (100). Interestingly, while medically managed patients often experienced functional decline from listing to transplant, patients supported by durable LVAD showed improved functionality during the same period (99). This provides indirect evidence of superior rehabilitation and conditioning with dMCS use.

Advancements in durable LVAD technology, particularly with HeartMate 3, have yielded encouraging results. The MOMENTUM 3 trial (10, 101) and ELEVATE registry (102) reported 2-year survival rates for HeartMate 3-supported patients ranging from 79% to 83.4%, nearing post-transplant survival rates. Furthermore, the 5-year Kaplan–Meier estimate of survival to transplant, recovery, or LVAD support free of debilitating stroke or reoperation to replace the pump in the HeartMate 3 was 54.0% (vs. 29.7% in the HeartMate II group, *p* < 0.001). Overall Kaplan–Meier survival at 5 years was 58.4%, compared to 43.7% with the HeartMate II (*p* = 0.003) (11), reaching 76% in patients under 50 years, with a lower incidence of serious adverse events compared to previous durable LVADs (103). Nonetheless, HF and device-related infections remain the leading causes of adverse events, morbidity, and mortality with fully magnetically centrifugal flow pump LVADs (11, 102). Careful patient selection and continuous assessment are crucial not only for optimizing waitlist outcomes but also for improving post-transplant success in patients bridged with the HeartMate 3. Factors such as advanced age, ischemic cardiomyopathy, renal dysfunction, obesity, and pulmonary hypertension have been associated with higher 1-year post-transplant mortality in patients supported by HeartMate 3 (104). Further studies focusing on long-term outcomes in patients bridged with fully magnetically levitated technology are needed to refine candidate criteria and enhance both pre- and post-transplant survival.

Finally, dMCS implantation has been associated with allosensitization in HT candidates (99, 105–108). However, unlike medically managed patients, allosensitization in dMCS-supported patients did not predict post-transplant mortality (106). Indeed, panel reactive antibody levels in these patients have been observed to rise early during support and decline over time leading to the hypothesis that the formation of pseudointima may reduce device-related immune activation (109). Device explantation may help mitigate inflammation and further lower panel reactive antibody levels, potentially rendering sensitization a transient phenomenon.

The use of MCS in older patients remains an area of permanent debate. Age correlates with higher in-hospital mortality (110), with a sharp increase after 72 years in those with AMI-CS under MCS (111). While age itself should not preclude MCS, frailty and comorbidities should be carefully assessed to better guide clinical management. Moreover, the optimal timing for the initiation of MCS remains difficult to determine in real-world practice. After an initial approach that includes optimizing medical treatment, the choice of further therapies should be tailored to the degree of hemodynamic support needed and availability of options. The use of vasoactive agents can complicate outcomes, and careful monitoring of catecholamine use and the vasoactive-inotropic score can help predict prognosis and guide MCS decisions (112). The goals of MCS should be clearly defined prior to implementation, emphasizing hemodynamic stabilization, decongestion and the restoration of systemic perfusion, while minimizing complications.

The controversy surrounding recipient age limits in HT arises from discrepancies between chronological and physiological age, variations in organ availability, and differing practices among transplant centers. Traditionally, older age has been considered a risk factor due to associations with increased comorbidities and a higher likelihood of post-transplant complications compared to younger recipients (113–115). As a result, many transplant centers establish a relative age cut-off, beyond which patients may not be considered HT candidates, with alternative options including LVAD therapy as DT (if eligible), destination inotrope therapy, or palliative care. However, successful survival outcomes in older patients at several transplant centers have led to increased consideration of this population for HT candidacy (114, 116). Interestingly, recent data indicate a 110% increase in HT among patients aged 50–64 (95, 116, 117); the increased listing of older adult candidates has led to a corresponding increase in the HT rate for these candidates, this trend is most pronounced in patients over 65 years old who went from a HT rate of 74.3 per 100 waitlist years in 2015 to 132.2 in 2019 (116).

With the evolving profile of transplant candidates, older patients are increasingly being considered for tMCS as a BTT. A small study by Paghdar et al. (95) focused on patients aged 50 or older [median age at HT was 63 (58–68)] with significant comorbidities who were supported with the Impella 5.5 device as BTT. The study demonstrated favorable survival outcomes with minimal complications, suggesting that tMCS may be a viable option in selected older candidates. However, further research is needed to better define the role of tMCS in patients over 65 years, a population that remains underrepresented in current evidence. Risk stratification in this group should go beyond chronological age to carefully account for comorbidity burden, support indication, and anticipated duration of therapy (95).

Despite the growing use of MCS as a BTT, most of the evidence supporting its effectiveness comes from registry data. To gain more reliable insights, it would be essential to conduct long-term randomized controlled trials to strengthen evidence-based clinical approaches and ultimately improve patient outcomes.

## 7 Interplay between MCS and HT allocation systems

Allocation systems are pivotal in the clinical decision process for HT candidates, carefully balancing the urgent medical needs of patients with the limited availability of donor organs. In many regions, HT allocation systems are based on urgency and compatibility factors, and designed to prioritize patients who are at the highest risk of mortality, which generally includes those supported by tMCS (81, 82, 118–120). Despite a continuous increase in the number of HT performed in recent years, the mismatch between organ demand and supply persists due to an increasing advanced HF population (9, 82, 121).

Previous reports showed that the use of tMCS as a direct BTT was not common around the world (81, 122), although this trend has been changing recently as new allocation systems tend to prioritize patients under tMCS (120, 123, 124). In countries like Spain (82, 125), with one of the highest rates of HT *per capita*, economic restrictions in accessing dMCS and timely donor availability make tMCS a relatively common method for BTT. In Spain, the highest level of priority on the waiting list is granted to patients on biventricular tMCS (VA-ECMO or biventricular Centrimag), followed by temporary LVAD (Impella 5.5 or LVAD Centrimag, but not Impella CP or IABP) and patients with refractory arrhythmic storm. Patients on dMCS are not prioritized, unless they have device-related complications (82). The global cumulative rate of HT in patients treated with tMCS listed for emergent transplant in Spain was 85% and showed an increasing tendency in the last few years, reflecting changes in donor allocation policies. Urgent HT represents more than one-third of the total transplants performed every year (125) and 1-year post-transplant survival was reported at 76% (82), with excellent transplant efficiency demonstrated by a median waiting time of six days for the higher urgency candidates.

The Eurotransplant coalition facilitates organ exchange among eight countries (119), prioritizing high-urgency HT candidates on inotropes, tMCS, or dMCS with device-related complications (126, 127). The waitlist mortality for HT candidates in the Eurotransplant network has significantly decreased over the past decade, reflecting improved organ utilization combined with the broadening donor pool and the utilization of durable LVADs as BTT (128). Alternatively, the French model employs a score-based allocation system, balancing urgency with donor-recipient compatibility (127), while the United Kingdom (UK) distinguishes “super-urgent” patients under tMCS (excluding IABP) or those with criteria for urgent transplant not suitable for durable LVAD (126). French, UK and Eurotransplant systems also do not prioritize stable durable LVAD patients.

In the United States of America (USA), policy revisions in the UNOS system in 2018 shifted the priority towards tMCS patients, leading to a significant reduction in the use of dMCS as BTT, from 29% to 5% between 2014 and 2021 (124, 129, 130). This change has shifted focus towards patients supported by tMCS (124, 131), granting them higher priority over those with stable dMCS, who are now classified as status 4 (out of 6 status levels) (120, 126). If complications during dMCS support arise they are upgraded to status 3 or status 2 in case of device malfunction. Prior to this policy change in the USA, stable durable LVAD patients were classified as status 1B (2 out of 3 status levels), with device-related complications allowing an upgrade to status 1A (126).

These allocation criteria are primarily driven by the prioritization of sicker patients and reflect the improved outcomes achieved in the last decade with dMCS, positioning durable LVADs more as a BTC or DT option rather than as a direct BTT. These changes in allocation have prompted transplant programs to adjust their practices in favor of tMCS to elevate candidate status and reduce waiting times for patients bridged to HT, which may raise ethical concerns.

The best allocation system remains an ongoing debate worldwide. To ensure equity, maximize clinical efficacy, and minimize organ wastage, continuous evaluation and refinement of allocation protocols are imperative.

## 8 Current gaps and challenges in the field

Despite the significant advances in MCS technology, several gaps in the field remain that warrant further investigation.

One major challenge is the management of patients with small left ventricles, such as those with restrictive or hypertrophic cardiomyopathies (132–135). These patients are at high risk for adverse outcomes but are often not candidates for conventional therapies that benefit patients with HF, presenting unique challenges in hemodynamic management and device selection. The small left ventricle size limits the ability to implant durable LVADs, which are designed for larger ventricles. Moreover, patients with these pathologies may exhibit biventricular dysfunction, which complicates the decision to use MCS devices as BTT, since biventricular assist devices carry higher risks and complications (136). Similar difficulties are faced when managing the growing population of adults with congenital heart disease because of the complex anatomic and physiologic features that characterize this heterogeneous group of patients, contributing to long waiting times and poor transplant outcomes (137). To address these gaps, some HT allocation systems prioritize patients with restrictive cardiomyopathies and congenital heart disease (71). Another related gap not completely met is right ventricular failure (138), difficult to treat and with unsatisfactory results with the devices currently available and an important cause of morbidity and mortality after implantation of durable LVADs. Emerging approaches, such as using a dual configuration HeartMate 3 pump for biventricular support (139, 140) or a redefined total artificial heart (141), show promising results but require further study. While there are several publications in the literature that discuss these issues, the available evidence remains insufficient to formulate comprehensive recommendations, and these gaps continue to represent significant challenges in the field.

Several challenging situations can arise during the care of patients with MCS. Therefore, the management of these devices requires a multidisciplinary approach integrated within other therapeutic interventions, such as pharmacotherapy and lifestyle modifications. Advancements in device design and automation can improve management but also introduce new challenges related to device operation, compatibility and software updates (142).

## 9 Future directions and research

There are significant opportunities to enhance the management of CS and chronic advanced HF, as well as to further develop MCS technologies. One promising and underexplored area is the use of ambulatory support devices—such as axillary or subclavian IABPs and dischargeable Impella devices (e.g., NCT05291884)—which may enable physical rehabilitation for patients awaiting HT (143, 144). These strategies could reduce complications, lower hospital costs, and potentially support pharmacologically mediated myocardial recovery.

Innovative therapies aimed at reverse remodeling, such as left ventricular volume reshaping, have shown potential in delaying the need for MCS in select ambulatory patients with reduced ejection fraction (145, 146). These interventions may offer a viable BTT—or even BTR—approach in well-selected individuals. In parallel, growing evidence supports the concept of myocardial recovery through early implementation of less invasive MCS devices (147). This highlights the importance of developing tools to identify HF reversibility prior to MCS implantation, optimizing unloading strategies with guideline-directed medical therapy, and implementing robust monitoring protocols to assess and support recovery.

Despite remarkable advances, the demand for more refined dMCS systems continues to grow. Future dMCS devices are expected to be less invasive, easier to implant, and more physiologically adaptive. Key developments under investigation include fully implantable pumps with wireless energy transfer systems to eliminate driveline infections, and improved biomaterials designed to reduce thrombotic risk and minimize anticoagulation needs (147–149). Artificial intelligence and machine learning are poised to transform MCS management by enabling predictive modeling, optimizing device settings, and personalizing therapy. Patient-specific computational simulations and phenotypic profiling are also emerging as tools to improve preoperative planning and long-term management (147).

Cross-disciplinary collaboration among clinicians, engineers, and industry stakeholders will be crucial to sustaining innovation in dMCS. Together, these advances aim to address current limitations, improve outcomes, and enhance the quality of life for patients with advanced HF.

## 10 Limitations

This review has several important limitations. As a narrative, non-systematic overview, it does not provide an exhaustive or comprehensive synthesis of all available evidence on MCS as a BTT, and some relevant data or studies may not have been included. The review focuses on the main devices currently used, their typical indications, and general outcomes. Due to the heterogeneity of patient populations, device types, and reporting standards across the literature, it is challenging to conduct quantitative analyses regarding survival and outcomes. Thus, we reported major findings but did not perform a comparative analysis or include data on MCS use after HT, which was beyond the scope of our review. Inconsistent reporting of important variables—such as duration of support while on the waitlist—hinders interpretation of outcomes. Additionally, recent changes in organ allocation systems, such as the prioritization of tMCS over stable durable LVADs, further complicate comparisons between these strategies for bridging, particularly with newer devices. Most of the available data are derived from observational studies, and reported outcomes are often conditioned by regional and institutional practices, including the selection and availability of specific device types. Finally, the lack of standardized reporting and potential publication bias further limit the ability to draw robust, generalizable conclusions from the available data and make specific recommendations.

## 11 Conclusions

The use of MCS as a BTT has revolutionized the management of end-stage HF. This review has examined its expanding role in HT candidates, focusing on clinical indications for device selection, the decision-making process, and outcomes associated with both short- and long-term MCS use. Available evidence suggests that dMCS improves waitlist survival and post-transplant outcomes compared to tMCS. Furthermore, patients bridged with dMCS have comparable post-transplant survival to those transplanted directly, reinforcing the value of durable devices in enhancing patient prognosis. However, trends in organ allocation increasingly prioritize the sickest patients requiring tMCS over those supported by stable LVADs. In this regard, recent devices such as the Impella 5.5 have demonstrated promising early results as BTT, and ongoing larger studies with long-term follow-up will help to better define their appropriate clinical indications and patient selection. The optimal allocation system remains a topic of ongoing debate, requiring a balance between urgency-based models that aim to reduce waitlist mortality and outcome-focused strategies that prioritize post-transplant survival, all within the constraints of national policies and resource availability. Despite technological advances, challenges persist in optimizing device selection, managing complications, and ensuring equitable allocation. Addressing these gaps through continued innovation and more personalized treatment approaches will be essential for improving device efficacy, safety, and quality of life for HT candidates. Future research should focus on refining allocation systems and overcoming current limitations to further enhance outcomes in this promising and dynamic field.
